# ISGF3 with reduced phosphorylation is associated with constitutive expression of interferon-induced genes in aging cells

**DOI:** 10.1038/s41514-018-0030-6

**Published:** 2018-11-15

**Authors:** Mari Yamagami, Motoyuki Otsuka, Takahiro Kishikawa, Kazuma Sekiba, Takahiro Seimiya, Eri Tanaka, Tatsunori Suzuki, Rei Ishibashi, Motoko Ohno, Kazuhiko Koike

**Affiliations:** Department of Gastroenterology, Graduate School of Medicine, The University of Tokyo, Tokyo, 113-8655 Japan

## Abstract

During cellular aging, many changes in cellular functions occur. A hallmark of aged cells is secretion of inflammatory mediators, which collectively is referred to as the senescence-associated secretory phenotype (SASP). However, the mechanisms underlying such changes are unclear. Canonically, the expression of interferon (IFN)-stimulated genes (ISGs) is induced by IFNs through the formation of the tripartite transcriptional factor ISGF3, which is composed of IRF9 and the phosphorylated forms of STAT1 and STAT2. However, in this study, the constitutive expression of ISGs in human-derived senescent fibroblasts and in fibroblasts from a patient with Werner syndrome, which leads to premature aging, was mediated mainly by the unphosphorylated forms of STATs in the absence of INF production. Under homeostatic conditions, STAT1, STAT2, and IRF9 were localized to the nucleus of aged cells. Although knockdown of JAK1, a key kinase of STAT1 and STAT2, did not affect ISG expression or IFN-stimulated response element (ISRE)-mediated promoter activities in these senescent cells, knockdown of STAT1 or STAT2 decreased ISG expression and ISRE activities. These results suggest that the ISGF3 complex without clear phosphorylation is required for IFN-independent constitutive ISG transcription in senescent cells.

## Aging and inflammation: aging cells express interferon-stimulated genes in a non-canonical pathway

Aging cells express many kinds of inflammation-related genes called SASP (senescence-associated secretary-phenotype), which are involved in aging-associated phenotypes. However, the underlying molecular mechanisms how such inflammatory gene expression is induced in aging cells are unclear. A team led by Motoyuki Otsuka at the University of Tokyo found that using senescent human fibroblasts interferon-stimulated genes do not express in a canonical interferon-related intracellular signaling pathway. Normally, interferon-stimulated genes are expressed through the phosphorylation of STAT proteins triggered by interferon stimulation. In contrast, in senescent cells, interferon-stimulated genes were highly expressed without interferon stimulation and the representative STAT phosphorylation was not induced. These findings indicate that the interferon-stimulated genes in aging cells are expressed in a mechanism different from a canonical interferon-related pathway. Further research into these phenomena may develop a way to intervene the senescence-associated phenotypes in aging.

## Introduction

Aging is inevitable and leads to various pathologies. However, the fundamental mechanisms underlying the age-related changes in cellular functions are unclear, and this is the main barrier to the development of strategies to prevent age-associated pathologies. Although cells undergoing aging, or senescent cells, display profound phenotypic changes, a hallmark of aged cells is the secretion of inflammatory mediators, such as interleukins (ILs); this is referred to as the senescence-associated secretory phenotype (SASP).^1,2^ Although the inflammatory response linked to the SASP is considered to underlie many age-related phenomena, the mechanisms underlying the regulation of the SASP remain incompletely understood.

Canonically, stimulation of IFN receptors by IFN activates the Janus kinases Jak1 and Tyk2. Subsequently, signal transducer and activator of transcription 1 (STAT1) and STAT2 are phosphorylated, promoting the formation of Stat1–Stat2 heterodimers, which associate with IFN regulatory factor 9 (IRF9) to form interferon (IFN)-stimulated gene factor 3 (ISGF3). ISGF3 translocates into the nucleus and binds to the specific promoter elements known as IFN-stimulated response elements (ISREs), leading to the transcription of IFN-stimulated genes (ISGs). In this canonical JAK-STAT paradigm, there is a strict correlation between the activities of STATs and their tyrosine phosphorylation.

In addition to the canonical JAK-STAT pathway, recent reports have assigned important tasks to unphosphorylated STATs.^3,4^ Strikingly, in the absence of detectable IFNs, constitutive ISG expression is mediated by the unphosphorylated ISGF3 complex, which comprises IRF9 and unphosphorylated STAT1 and STAT2, in some mouse and human organoids.^3^

In this study, we confirmed that normal human dermal fibroblasts (NHDFs) that underwent in vitro cellular aging after serial passages exhibit a higher level of expression of ISGs than lower passaged cells. However, IFNs, including IFNα and β, were not significantly upregulated in these cells. This finding led us to hypothesize that the aberrant expression of ISGs in senescent cells is mediated mainly by the unphosphorylated forms of STATs. We show that unphosphorylated STAT1 and STAT2 protein levels are increased in NHDFs after in vitro cellular aging as well as in fibroblasts from a patient with Werner syndrome, which leads to premature aging. Knockdown studies confirmed that the higher STAT1 and STAT2 protein levels, but not JAK1, are involved in the activities of ISREs in senescent cells. Thus, we concluded that the ISGF3 complex without clear phosphorylation is required for constitutive ISG expression in senescent cells under physiological conditions.

## Results

### Expression of senescence markers in passaged human dermal fibroblasts

**Fig. 1:**
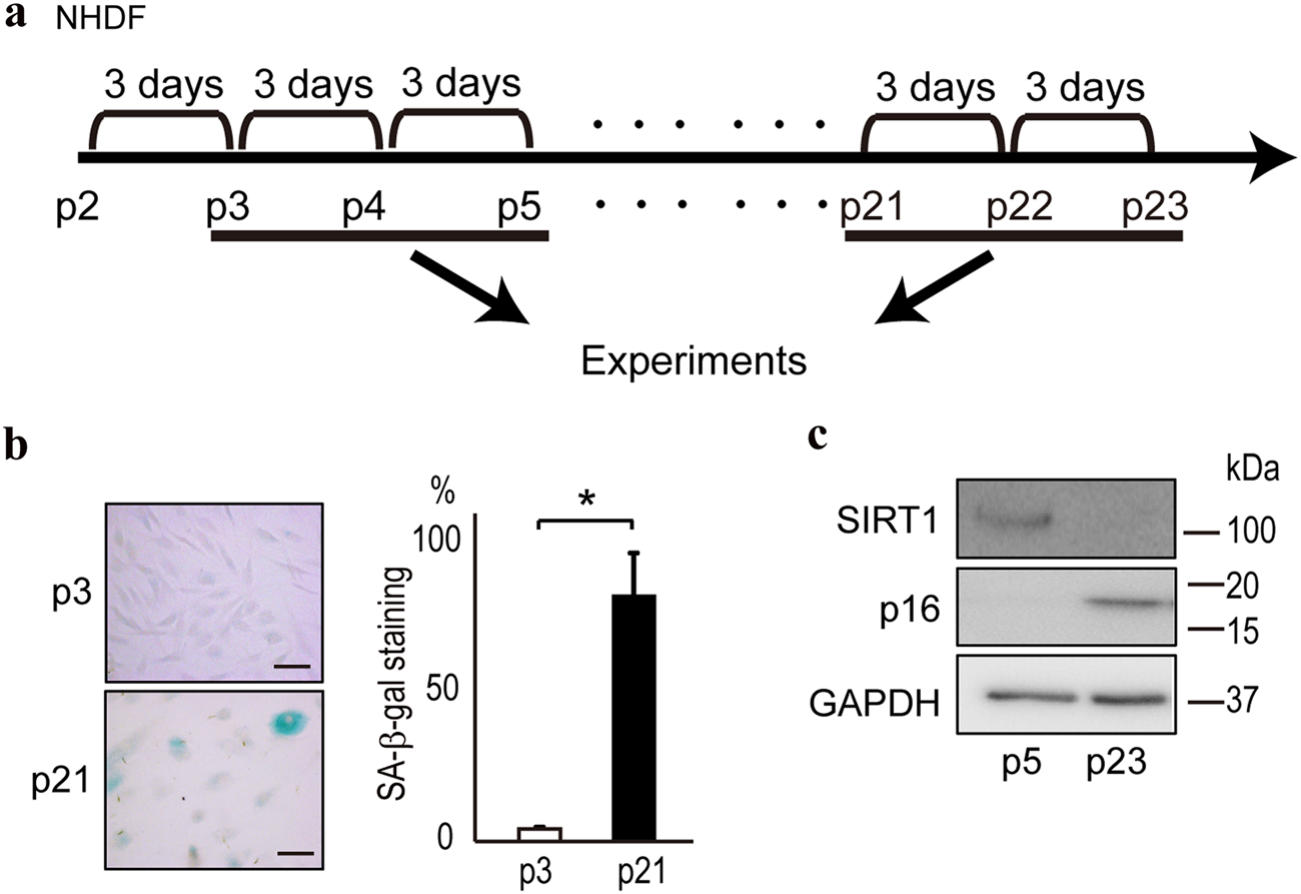
In vitro aging of NHDFs after serial passages. **a** NHDFs isolated from normal human dermal fibroblasts were obtained at passage 2. After starting the cultures, cells were passaged every 3 days. Early passage cells, passages 3 (p3) to 6 (p6); senescent cells, passage > 20. **b** Confirmation of the aging of NHDFs at later passages. SA-β-gal, a senescence marker, was stained in cells at passages 3 (p3) and 21 (p21). Staining was denser in cells at later passages. Representative results from three independent experiments are shown. Bar, 10 µm. Positive cells were determined by counting 20 cells in every fifth field of view from three experiments in each group. Data are expressed as means ± standard errors (s.e.). *p < 0.05. **c** Higher p16^INK4A^ protein levels and lower SIRT1 levels were detected in HNDFs at passage 23 (p23) compared with at passage 5 (p5), as determined by western blotting. Representative results from three independent experiments are shown

To determine whether NHDFs subjected to multiple passages show characteristics of aging, we examined the SA-β-gal expression level and the protein levels of representative senescence markers in NHDFs after passaging at 3-day intervals for 2 months (Fig. 1a). Compared with NHDFs passaged three times over circa 10 days, NHDFs subjected to a greater number of passages showed more intense staining for SA-β-gal, a marker of senescence (Fig. 1b), as well as more intense staining for 8-OHdG, a marker of DNA damage (Supplementary Figure 1). These aged cells exhibited increased expression of p16^INK4a^, another marker of senescence (Fig. 1c), and decreased expression of SIRT1, the level of which decreases with age (Fig. 1c). Thus, the passaged cells had the characteristics of aged cells, which is consistent with previous reports.^5,6^

### ISGs are upregulated in the absence of IFN production in senescent NHDFs

**Fig. 2:**
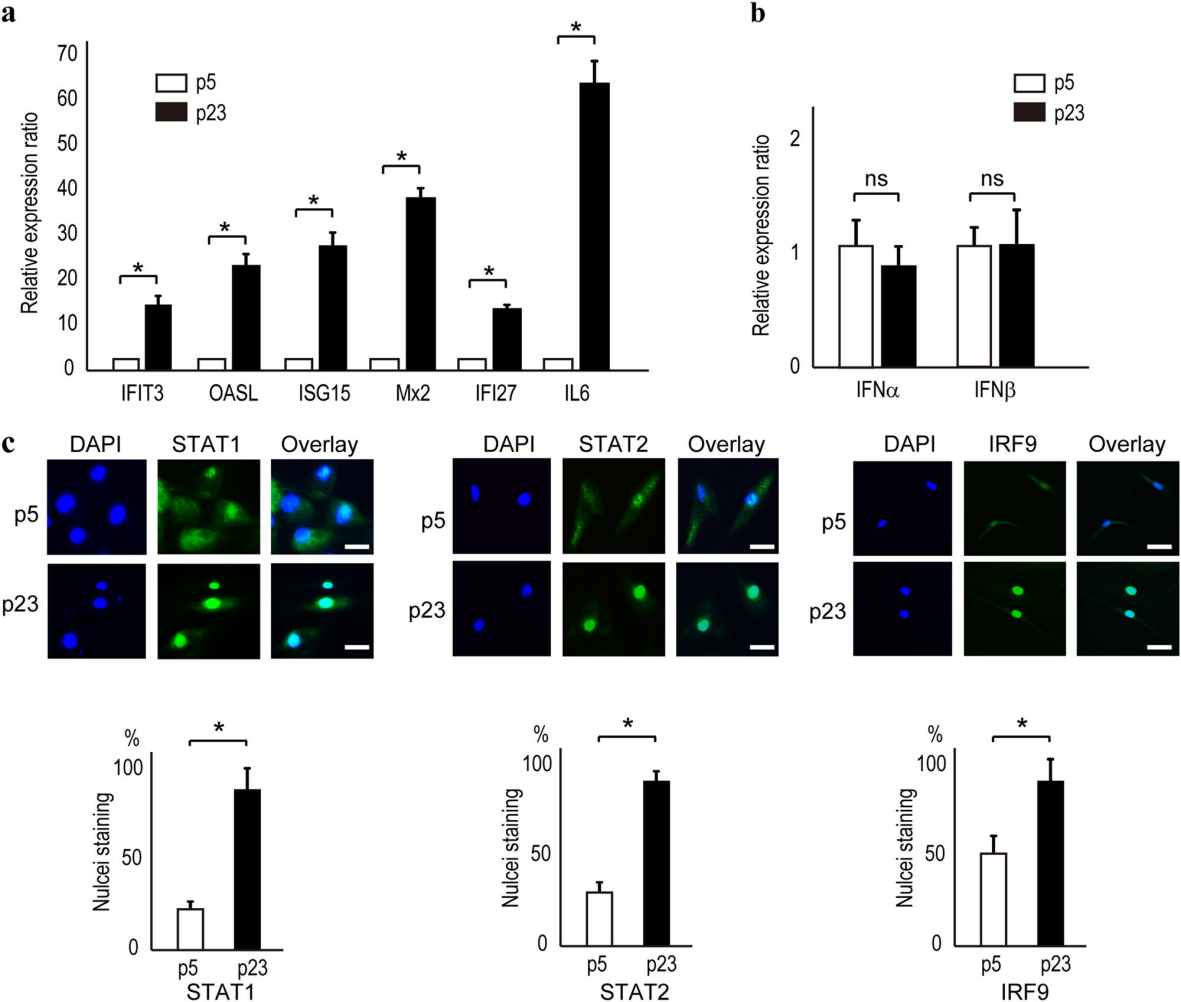
ISGs are upregulated in senescent NHDFs. **a** According to quantitative RT-PCR, ISG mRNA levels were significantly increased in NHDFs at passage 23 (p23) compared with passage 5 (p5). Data are expressed as means ± s.e. of triplicate results from two independent experiments. *p < 0.05. IL-6 was the positive control. **b** IFNα and IFNβ mRNA levels were comparable in NHDFs at passages 5 and 23 (p5 and p23). Data are expressed as means ± s.e. from two independent experiments performed in triplicate. *p < 0.05. **c** STAT1, STAT2, and IRF9 localized to the nucleus of NHDFs at passage 23 (p23). The STAT1, STAT2, and IRF9 protein levels were increased and their nuclear localization was enhanced in NHDFs at passage 23 (p23) compared with passage 5 (p5). Representative results from three independent experiments are shown. Bar, 10 µm. Positive nuclei were determined by counting 20 cells in every fifth field of view from three experiments in each group. Data are expressed as the means ± s.e. *p < 0.05

To evaluate the transcriptional profile of senescent cells, we determined the levels of mRNAs in cells at passages 5 and 23 using cDNA arrays. IL-6 expression was ~ 76-fold higher in cells at later compared with earlier passages (Supplementary Table 1 and Fig. 2a), which is consistent with previous reports that aged cells express SASP.^7,8^ The expression levels of several ISGs—ISG15, Mx, OASL, and IFITs—were also markedly increased in cells at later passages (Supplementary Table and Fig. 2a). However, IFNα, IFNβ, and IFNγ, which upregulate ISG transcription, were not significantly upregulated (Supplementary Table and Fig. 2b); this was confirmed with the primers used to detect upregulated IFNα and IFNβ production in Huh7 cells stimulated with polyI-polyC (pIpC), which induces IFN production (Supplementary Figure 2). The expression of ISGs is believed to be induced by the nuclear translocation of the tripartite transcription factor ISGF3, which is composed of IRF9 and the phosphorylated forms of STAT1 and STAT2, in the presence of IFNs. In the case of NHDFs, STAT1, STAT2, and IRF9 were localized to both the cytoplasm and the nucleus in early passage cells but exclusively to the nucleus in senescent cells (Fig. 2c). These results suggest that the transcription factor ISGF3, together with STAT1 and STAT2, translocates into the nucleus, which leads to significant, and IFN-independent, upregulation of ISG expression in senescent cells.

### Unphosphorylated ISGF3 drives ISG expression in aged cells

**Fig. 3:**
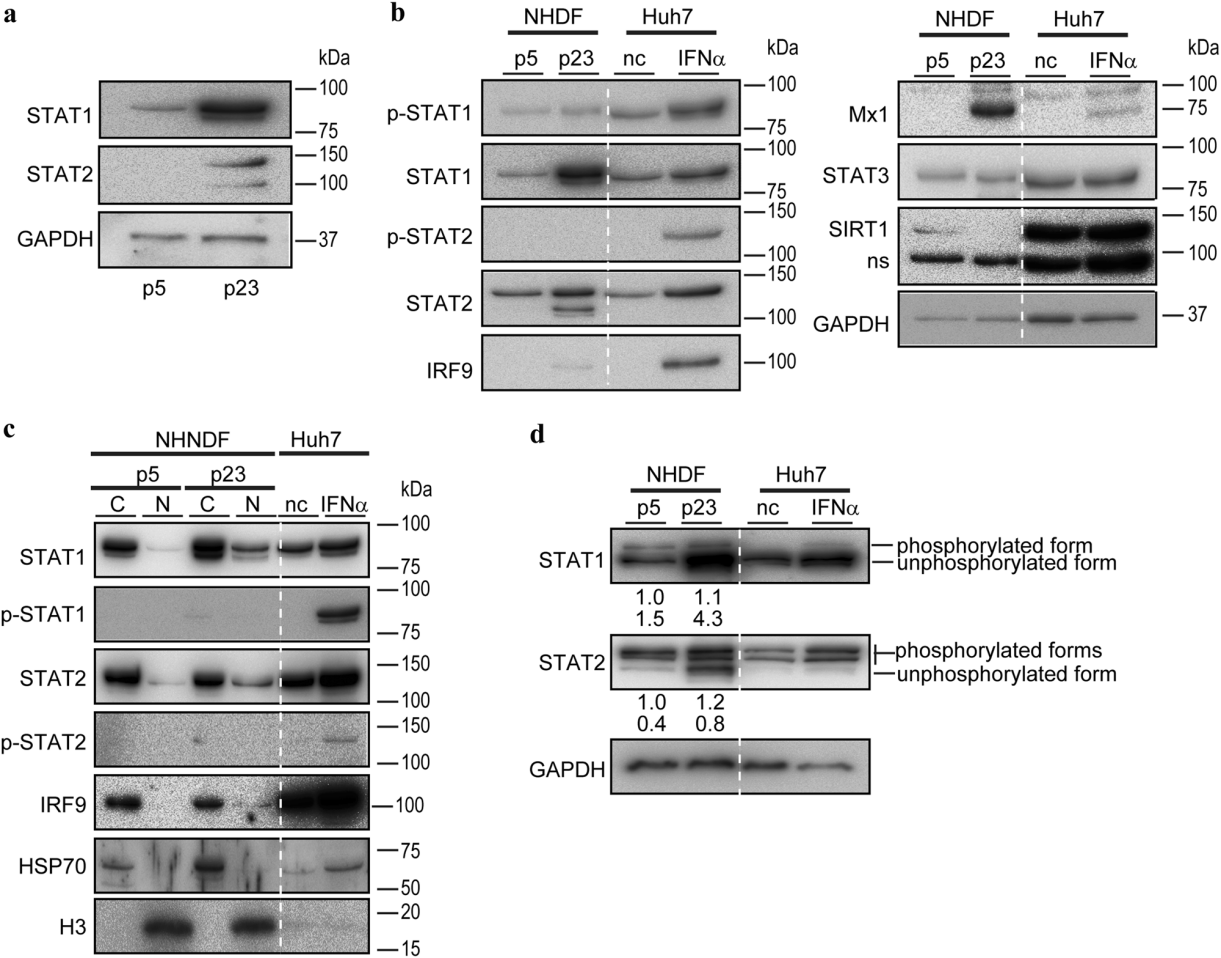
Unphosphorylated STAT levels are increased in the nucleus of senescent NHDFs. **a** STAT1 and STAT2 protein levels were increased in NHDFs at passage 23 (p23), compared with passage 5 (p5). Representative results from three independent experiments are shown. **b** Phosphorylated STAT1 and STAT2 levels were not increased in NHDFs at passage 23 (p23). STAT1, STAT2, IRF9, and Mx1 protein levels were increased; the STAT3 protein level was unchanged; and the SIRT1 protein level was decreased in the nucleus of NHDFs. Cell lysates from Huh7 cells treated with IFNα for 12 h were used as controls. Representative results from three independent experiments are shown. nc negative control, ns nonspecific bands. **c** Unphosphorylated STAT1 and STAT2 protein levels were increased in the nucleus of NHDFs at passage 23 (p23) compared with passage 5 (p5). Cytoplasmic (C) and nuclear (N) fractions were extracted from NHDFs and subjected to western blotting. HSP70 and histone H3 proteins were used as controls for cytoplasmic and nuclear proteins, respectively. Whole-cell lysates from Huh7 cells treated with IFNα for 12 h were used as controls. Representative results from three independent experiments are shown. nc negative control. **d** The levels of all phosphorylated forms of STAT1 and STAT2 were determined by phos-tag gel analyses. The levels of unphosphorylated STAT1 and STAT2 proteins were increased in NHDFs at passage 23 (p23) compared with passage 5 (p5). Lysates of Huh7 cells treated with IFNα for 12 h were used as controls. Representative results from three independent experiments are shown. nc negative control. The numbers below the panel indicate the band intensities for the phosphorylated forms (upper) and nonphosphorylated forms (lower)

Consistent with the finding of increased STAT1 and STAT2 mRNA levels (GEO accession #GSE107483 and Supplementary Table 1), the protein levels of STAT1 and STAT2 were higher in senescent cells (Fig. 3a). Importantly, the key phosphorylated forms of STAT1 (phosphorylation at tyrosine 701) and STAT2 (phosphorylation at tyrosine 690) were induced in Huh7 cells after IFNα treatment, which is consistent with previous reports^9,10^ (Fig. 3b). However, in the senescent NHDFs, the levels of the phosphorylated forms of STAT1 and STAT2 were not increased compared with those in early passage cells (Fig. 3b), although the STAT1, STAT2, and IRF9 protein levels were increased. The expression level of Mx1, a representative ISG, was increased in the senescent cells and in Huh7 cells after IFN treatment, and the SIRT1 protein level was decreased in senescent cells, confirming that the NHDFs entered a senescent state at passage 23 (Fig. 3b). The expression level of STAT3, a STAT related to the IL-6 pathway but not the IFNα and β pathways, was unchanged (Supplementary Table 1 and Fig. 3b). These results suggest that, in senescent cells, induction of ISGs is independent of IFN production and the phosphorylation of STAT1 and 2, upstream elements of the IFN signaling pathway.

Although the levels of the phosphorylated forms of STAT1 and STAT2 were not significantly increased in the senescent cells, translocation of these proteins into the nucleus was detected by immunocytochemistry (Fig. 2c). Thus, we next examined the intracellular distribution of STAT1 and STAT2 proteins by fractionating the cytoplasmic and nuclear proteins of the early passage and senescent cells. The nuclear STAT1 and STAT2 protein levels were significantly increased in senescent cells (Fig. 3c), whereas, despite the marked increases in their levels in Huh7 cells after IFN treatment, the phosphorylated forms of STAT1 and STAT2 were not detected (Fig. 3c).

To examine whether non-phosphorylated STAT1/2 formed a complex with IRF9 in senescent cells, we performed an immunoprecipitation assay based on the precipitation of IRF9. Increased amounts of STAT1/2 were detected in IRF9-related complexes in senescent cells (Supplementary Figure 3) compared with earlier passaged cells, while these proteins were not detected by representative phosphor-specific antibodies (Tyr 701 in STAT1 and Tyr 690 in STAT2). Moreover, a chromatin immunoprecipitation (ChIP) assay using anti-IRF9 antibodies confirmed that more IRF9-related protein complexes existed at the promoter regions of *ISRE* genes (e.g., *IFI27* and *Mx1*) in senescent cells (Supplementary Figure 4). These results suggest that a protein complex consisting of STAT1/2 and IRF9 associates with the promoter regions of some *ISRE* genes in senescent cells.

Next, we evaluated phosphorylation at residues other than Tyr 701 in STAT1 and Tyr 690 in STAT2 and assessed the levels of the phosphorylated forms of STAT1 and STAT2 in senescent cells by phos-tag assay.^11^ While IFN treatment resulted in increased levels of the phosphorylated forms of STAT1 and STAT2 in Huh7 cells (Fig. 3d), these forms were increased in senescent cells, although some phosphorylated forms of STAT1 and STAT2 were also detected in both early passage and senescent cells (Fig. 3d). These results suggest that unphosphorylated STAT1 and STAT2 translocate into the nucleus independently of IFN production under physiological conditions in senescent cells.

### ISG expression and unphosphorylated ISGF3 levels are increased in fibroblasts from a patient with Werner syndrome

**Fig. 4:**
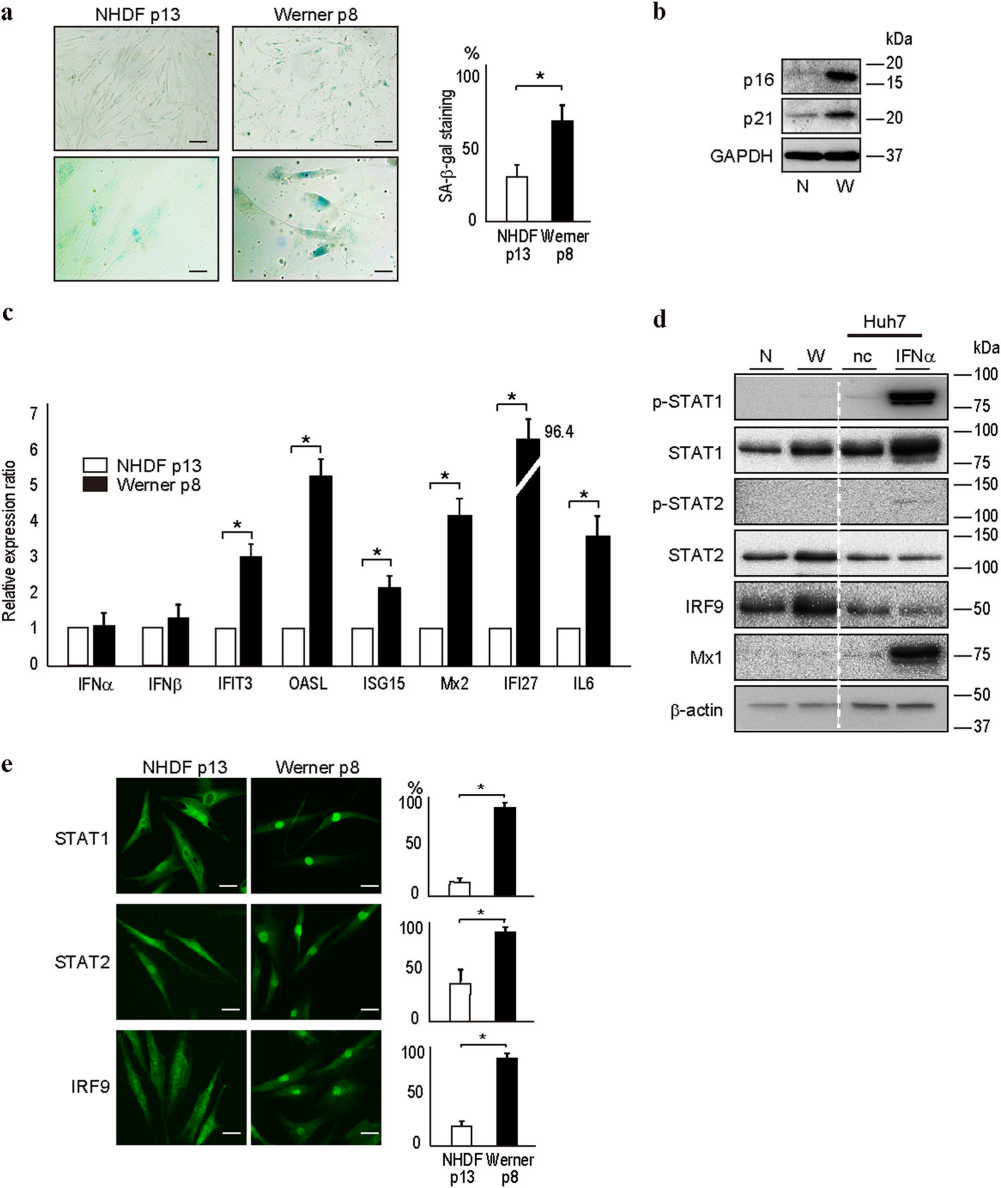
Unphosphorylated STAT1 and STAT2 levels are increased in fibroblasts from a patient with Werner syndrome. **a** SA-β-gal staining in fibroblasts from a patient with Werner syndrome at passage 8 (p8) and in NHDFs at passage 13 (p13). SA-β-gal staining was more intense in fibroblasts at passage 8 compared with NHDFs at passage 13. Representative results from three independent experiments are shown. Bars in upper and lower panels, 50 and 10 µm. Positive cells were determined by counting 20 cells in every fifth field of view from three experiments in each group. Data are expressed as the means ± s.e. *p < 0.05. **b** p16^INK4A^ and p21 protein levels were higher in fibroblasts from a patient with Werner syndrome, as determined by western blotting. N and W indicate NHDFs at passage 13 (p13) and fibroblasts from a patient with Werner syndrome at passage 8 (p8), respectively. Representative results from three independent experiments are shown. **c** The ISG mRNA level was higher in fibroblasts from a patient with Werner syndrome at passage 8 (p8) than in NHDFs at passage 13 (p13), as determined by quantitative RT-PCR. Data are expressed as means ± s.e. of triplicate results from two independent experiments. *p < 0.05. **d** Unphosphorylated STAT1 and STAT2 levels were higher in fibroblasts from a patient with Werner syndrome at passage 10 (W) than in NHDFs at passage 13 (N). Lysates from Huh7 cells treated with IFNα for 12 h were used as positive controls. nc negative control. Representative results from three independent experiments are shown. **e** STAT1, STAT2, and IRF9 localized to the nucleus of fibroblasts from a patient with Werner syndrome. The STAT1, STAT2, and IRF9 protein levels were increased and their nuclear localizations were enhanced in fibroblasts from a patient with Werner syndrome at passage 8 (p8) compared with those in NHDFs at passage 13 (p13). Representative results from three independent experiments are shown. Bar, 10 µm. Graphs on the right indicate the percentages of the cells with stronger staining in the nucleus compared to the cytoplasm by counting five different views. Data are expressed as the means ± s.e. *p < 0.05

To confirm the above findings in other senescent human cells, we examined fibroblasts isolated from a patient with Werner syndrome, which is an autosomal-recessive disorder characterized by the premature appearance of features of normal aging in young adults.^12,13^ Fibroblasts from a patient with Werner syndrome showed SA-β-gal staining at passage 8 compared with NHDFs at passage 13 (Fig. 4a) as well as higher levels of the senescence markers, p16^INK4a^ and p21 (Fig. 4b). Similar to senescent NHDFs, fibroblasts from a patient with Werner syndrome also showed higher expression levels of the ISGs, IFIT3, OASL, ISG15, Mx2, and IFIT27, even at earlier passages (Fig. 4c). However, similar to senescent NHDFs, phosphorylated STAT1 and STAT2 were not detected, whereas the level of unphosphorylated ISGF3 (comprising STAT1, STAT2, and IRF9) was increased (Fig. 4d). In addition, there was increased translocation of STAT1, STAT2, and IRF9 into the nucleus in fibroblasts from a patient with Werner syndrome at passage 8 compared with NHDFs at passage 13 (Fig. 4e). Therefore, the increased ISG expression in cells from a patient with Werner syndrome may be independent of phosphorylated STAT1 and STAT2, similar to the case in senescent NHDFs.

### ISRE activity in senescent NHDFs is independent of JAK1

**Fig. 5:**
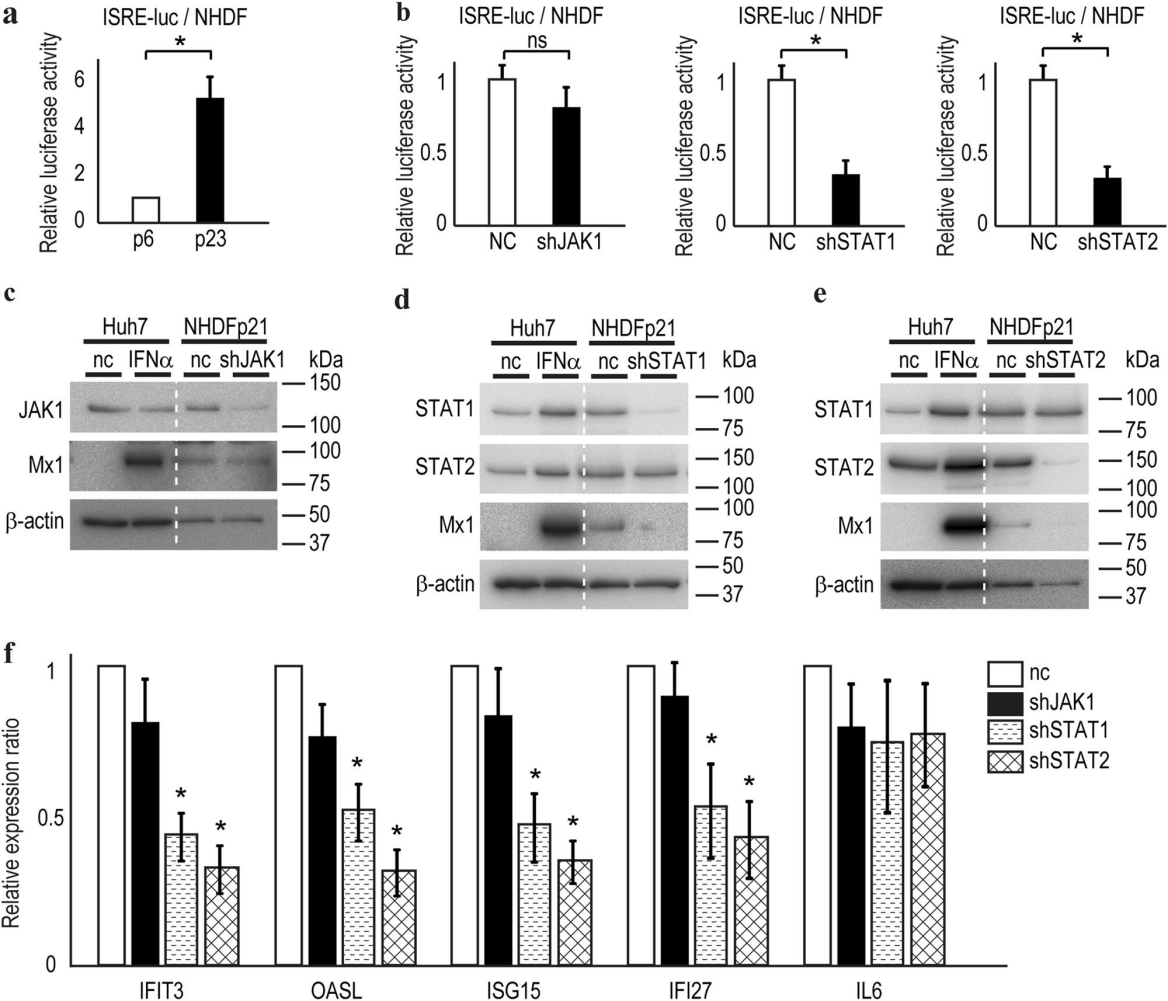
JAK1 is not related to ISRE promoter activity in senescent cells. **a** ISRE activities are higher in NHDFs at passage 23 (p23) compared with passage 6 (p6). ISRE-driven luciferase expression constructs were transiently transfected, and a dual luciferase assay was performed. Data are expressed as means ± s.e. from two independent experiments performed in triplicate. *p < 0.05. **b** JAK1 knockdown did not affect ISRE activity in NHDFs at passage 24, but STAT1 or STAT2 knockdown decreased the activity of ISRE. Data are means ± s.e. from two independent experiments performed in triplicate. *p < 0.05. **c−e** JAK1 knockdown did not affect the Mx1 protein level (**c**), but STAT1 or STAT2 knockdown decreased the Mx1 protein level (**d**, **e**). Representative results from three independent experiments are shown. **f** The ISG mRNA level was reduced by STAT1 or STAT2 knockdown but not by JAK1 knockdown in NHDFs at passage 24. IL6 was included as an unrelated gene. Data are expressed as the means ± s.e. from two independent experiments performed in triplicate. *p *<* 0.05

Because ISG induction in senescent cells may be independent of STAT1 and STAT2 phosphorylation, we next examined the involvement of JAK1, a key upstream kinase of the STAT1 and STAT2 signaling pathway. We used a transcriptional reporter system that mimics the IFN response pathway. This system comprises a luciferase reporter gene, expression of which is driven by multiple ISREs (ISRE-luc), together with lentiviral vectors expressing gene-specific short hairpin RNAs (shRNAs). ISRE-luc activity in Huh7 cells was induced by IFNα treatment (Supplementary Figure 5), and ISRE-luc activity was significantly higher in senescent than early passage NHDFs (Fig. 5a). In senescent cells, although both shSTAT1 and shSTAT2 decreased ISRE-luc activity, shJAK1 did not significantly affect the ISRE-luc activity (Fig. 5b). Western blotting confirmed the effects of the shRNA constructs on JAK1 in these assays (Fig. 5c). The expression of a representative ISG gene, *Mx1*, was unaffected by JAK1 knockdown in senescent cells (Fig. 5c). In contrast, shSTAT1 and shSTAT2 decreased the Mx1 protein level (Fig. 5d, e), indicating that both STAT1 and STAT2 are crucial for induction of ISG expression in senescent cells. Taken together, these data suggest that induction of ISG expression in senescent cells is independent of JAK1 and IFN but is dependent on STAT1 and STAT2 expression.

### STAT1 and STAT2 protein levels are increased in liver stellate cells from elderly patients

**Fig. 6:**
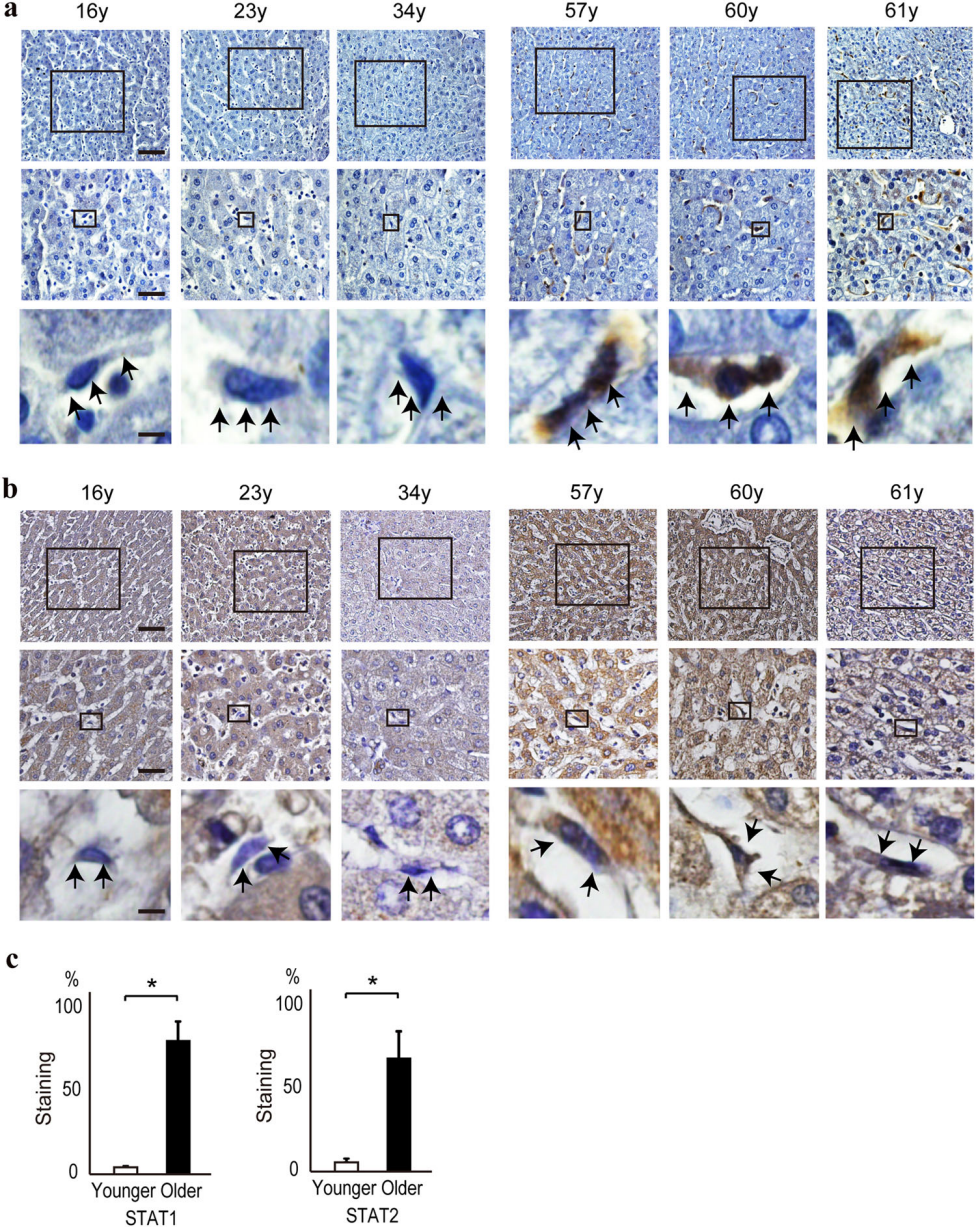
STAT1 and STAT2 levels are higher in liver stellate cells from senescent subjects. **a**, **b** STAT1 (**a**) and STAT2 (**b**) levels in human liver tissues were determined by immunohistochemistry. Staining was more intense in stellate cells in liver tissues from 57-, 60-, and 61-year-old persons than in those from 16-, 23-, and 34-year-old persons. The lower panels show enlargements of the boxed areas in the upper panels. Bars in top, middle, and lower panels: 1 mm, 100 µm, and 10 µm, respectively. Arrowheads indicate stellate cells. **c** Stellate cells with positively stained nuclei were determined by counting 20 cells in every fifth field of view from each patient sample. Data are expressed as means ± s.e. *p < 0.05

To confirm the above findings in vivo, we determined by immunohistochemistry the STAT1 and STAT2 levels in liver tissues from younger and older patients. In older patients, the STAT1 protein level was significantly increased in both the cytoplasm and the nucleus of stellate cells, but it was not increased in liver parenchymal cells (Fig. 6a). The STAT2 protein level was increased in liver parenchymal cells (Fig. 6b), and it was increased to a greater extent in stellate cells as well in the livers of older patients (Fig. 6b). These results suggest that the STAT1 and STAT2 protein levels are increased in some types of older human cells, likely proliferating cells, similar to in in vitro-aged cells.

## Discussion

Herein, we show that ISG expression is significantly increased in in vitro-aged NHDFs in a manner independent of IFN production but mediated by increased levels of apparently unphosphorylated STAT1 and STAT2 proteins. These results may illuminate the unique mechanisms of ISG expression in senescent cells under physiological conditions.

Classically, expression of ISGs is induced by the transcription factor ISGF3, which translocates into the nucleus after its formation via complexation of IRF9, phosphorylated STAT1, and phosphorylated STAT2. STAT phosphorylation is mediated by JAK1 in the presence of IFN. However, unphosphorylated ISGF3 also drives constitutive expression of ISGs independently of IFN.^3^ Aging is associated with the production of various cytokines (i.e., SASP, which underlies many age-related disorders).^1^ Our results show that the induction of ISG expression in senescent cells is independent of IFN production but dependent on increased levels of apparently unphosphorylated STAT1 and STAT2.

ISGs are critical in the response to viral infections.^14^ However, abnormally enhanced basal ISG expression is associated with adverse consequences in some viral infections.^15^ ISGs exert antitumor effects, but abnormally high basal expression of ISGs promotes tumor growth.^16^ Therefore, the effects of basal ISG expression may differ from those of ISG expression induced by IFN. Although whether the upregulated ISG expression in aging cells is associated with aging itself or with age-related pathological conditions needs to be clarified, the involvement of unphosphorylated ISGF3 in basal ISG expression in senescent cells may facilitate development of therapeutic interventions.

The induction of ISG expression in the senescent cells was independent of STAT1 and STAT2 phosphorylation, whereas the classical JAK-STAT paradigm involves a strict correlation between STAT activity and STAT tyrosine phosphorylation. Unphosphorylated STATs also play diverse roles.^3^ Additionally, modifications other than phosphorylation, such as acetylation^17^ or methylation,^18^ of STAT proteins may be involved in STAT-dependent ISG production. Whereas the overall STAT1 and STAT2 phosphorylation status was unchanged, it is possible that modifications of STAT1 and STAT2 other than phosphorylation are involved in the high expression levels of ISGs under physiological conditions in the absence of IFN production in senescent cells. The involvement of such post-translational modifications of STAT1 and STAT2, the mechanisms underlying the increased levels of their unphosphorylated forms, and their nuclear translocation in senescent cells remain to be clarified. Because unphosphorylated ISGF3 is involved in resistance to DNA damage,^19^ and given that unphosphorylated ISGF3 protects against genome instability in *Drosophila*,^20^ increased unphosphorylated ISGF3 in senescent cells may reflect increased DNA damage in these cells. The precise mechanisms and biological significance of increased unphosphorylated ISGF3 levels in senescent cells should therefore be determined in the future.

In this study, we used NHDFs at various passages, as well as fibroblasts from a patient with Werner syndrome, to examine the characteristics of human aging. However, these characteristics may differ among cell and/or organ types. For example, the magnitude of the age-related increase in the STAT2 level was lower in liver parenchymal cells than stellate cells. Also, no such increase in STAT1 level was observed in parenchymal cells. This may be because, under normal physiological conditions, liver parenchymal cells do not divide and may thus undergo gradual aging. Therefore, it would be interesting to examine cells under pathological conditions, such as chronic inflammation (which involves increased cell division) and to determine the timing of the phenomena described in this study in various cell types. Any differences detected may be related to susceptibility to age-related diseases. Furthermore, the biological significance of the increase in STAT1/2 in hepatic stellate cells during human aging detected in this study remains to be determined. Because these cells are involved in innate liver immunity,^21^ these phenomena may be related to the compromised immunity of elderly subjects. These possibilities should be evaluated using an in vivo model or other experimental conditions.

In summary, our findings reveal that, in senescent cells, the expression of ISGs is induced in an apparent STAT1 and STAT2 phosphorylation- and IFN production-independent manner. In addition to the SASP, other as-yet-undefined molecular events may occur in aging cells. Further elucidation of the roles of STAT1 and STAT2 may enable development of chemotherapeutic agents to prevent aging and/or age-related pathologies.

## Methods

### Cell lines

NHDFs at passage 2 were obtained from PromoCell (Heidelberg, Germany) and cultured in fibroblast basal medium supplemented with 2% fetal bovine serum, fibroblast growth factor, and insulin. TrypLE (Thermo Fisher, Waltham, MA) was used to dissociate the cells. Passages were performed approximately every 3 days. Fibroblasts from a patient with Werner syndrome were obtained from the JCRB Cell Bank at passage 7 and cultured in RAMPI1640 medium containing 20% fetal bovine serum; these cells were passaged similarly to NHDFs. Huh7 cells (control) were obtained from the American Type Culture Collection (ATCC, Manassas, VA) and were maintained in Dulbecco’s modified Eagle’s medium supplemented with 10% fetal bovine serum. The cells were incubated at 37 °C in an atmosphere containing 20% O_2_ and 5% CO_2_.

### SA-β-gal staining

SA-β-gal staining was performed using a Cellular Senescence Assay Kit (Chemicon International, Temecula, CA) according to the manufacturer’s protocol. Briefly, cells were washed in phosphate buffered saline (PBS), fixed for 10–15 min at room temperature in fixing solution, washed, and incubated at 37 °C (no CO_2_) with fresh SA-β-gal staining solution for 4 h. The cells were observed using an AX80 microscope (Olympus, Tokyo, Japan).

### Reagents

pIpC was purchased from Miltenyi Biotec (Auburn, CA). IFNα production in Huh7 cells was induced by adding 10 μg/mL pIpC to the medium; cells were collected after 24 h. Human recombinant IFNα was purchased from R&D Systems (Minneapolis, MN). Huh7 cells were treated with 100 U/mL IFNα for 12 h to prepare positive controls.

### Western blot analysis and antibodies

Western blotting was performed as described previously.^22^ Lysates were separated on 10–20% gradient sodium dodecyl sulfate-polyacrylamide gel electrophoresis (SDS-PAGE) gels, followed by electrical transfer to polyvinylidene difluoride membranes (GE Healthcare, Chicago, IL). After blocking with 5% skim milk, membranes were probed with the appropriate primary antibodies diluted in Immunoshot Reagent 1 (Cosmo Bio, Tokyo, Japan) overnight at 4 °C. The corresponding horse raddish peroxidase (HRP)-conjugated secondary antibodies (GE Healthcare) were next applied. Bound antibodies were detected using Immunostar LD reagents (Wako, Osaka, Japan). The following antibodies were used: anti-STAT1 (#14994, 1:1000), -STAT2 (#72604, 1:1000), -phosphor-STAT1 (Tyr701) (#9167, 1:1000), -phosphor-STAT2 (Tyr690) (#88410, 1:1000), -IRF9 (#76684, 1:1000), -Mx1 (#37849, 1:1000), -STAT3 (#12640, 1:1000), p21 (#2947, 1:1000), -SIRT1 (#9475, 1:1000), -HSP70 (#4876, 1:1000), -histone H3 (#4499, 1:1000), -JAK1 (#3344, 1:1000), and -HRP-conjugated β-actin (#12620, 1:2000) from Cell Signaling Technology (CST, Danvers, MA); anti-GAPDH (#H00002597; 1:500) from Abnova (Taipei, Taiwan); and anti-p16^INK4A^ (ab54210, 1:500) from Abcam (Cambridge, UK). Full, uncropped images are in the Supplementary files (Supplementary Figure 6 and 7). All blots were derived from the same experiment and were processed in parallel.

### RNA extraction, cDNA microarray, and quantitative RT-PCR

RNA was extracted using Isogen II (Nippon Gene, Toyama, Japan) according to the manufacturer’s protocol. cDNA microarray analyses were performed using cDNA oligo chips (Toray Industries, Tokyo, Japan). Data were deposited in the GEO database (accession number GSE107483). Quantitative RT-PCR (qRT-PCR) was performed as described previously.^22^

Briefly, total RNA was reverse-transcribed to cDNA using SuperScript III reverse transcriptase (Invitrogen, Carlsbad, CA). PCR was performed using FastStart Universal SYBR Green Master Mix with rox (Roche Diagnostics, Basel, Switzerland) with StepOnePlus Real-Time PCR instruments (Invitrogen).

All values were normalized to the mRNA level of the housekeeping gene, β-actin, the expression of which was unaffected by the number of cell passages according to a cDNA microarray. Relative expression was calculated according to the ΔΔC_T_ method as follows: ΔΔC_T_ = ΔC_Tsample_ − ΔC_Tβ-actin_. The primers used were as follows: IL6, Fw: 5′-ACCCCTGACCCAACCACAAAT-3′ and Rv: 5′-AGCTGCGCAGAATGAGATGAGTT-3′; IFNα, Fw: 5′-GACTCCATCTTGGCTGTGA-3′ and Rv: 5′-TGATTTCTGCTCTGACAACCT-3′; IFNβ, Fw: 5′- GTCACTGTGCCTGGACCATAG-3′ and Rv: 5′-GTTTCGGAGGTAACCTGTAAGTC-3′. IFIT3, Fw: 5′-AGAAAAGGTGACCTAGACAAAGC-3′ and Rv: 5′- CCTTGTAGCAGCACCCAATCT-3′; OASL, Fw: 5′- CCATTGTGCCTGCCTACAGAG-3′ and Rv: 5′-CTTCAGCTTAGTTGGCCGATG -3′, ISG15, Fw: 5′- CTCTGAGCATCCTGGTGAGGAA -3′ and Rv: 5′- AAGGTCAGCCAGAACAGGTCGT -3′; Mx2, Fw: 5′- CAGAGGCAGCAGACGATCAAC -3′ and Rv: 5′-TTGGTCAGGATACCGATGGTC-3′, and IFI27, Fw: 5′- TGCTCTCACCTCATCAGCAGT-3′ and Rv: 5′- CACAACTCCTCCAATCACAACT-3′.

### Immunofluorescence

Cells were seeded in four-well glass chambers (Iwaki, Shizuoka, Japan) manually coated with collagen. Cells were fixed with 4% paraformaldehyde in 1× PBS for 15 min at RT and permeabilized with 0.1% Triton-X in 1× PBS for 20 min at room temperature. Primary antibodies diluted 1:100 in Can Get Signal Immunostaining Solution A (Toyobo, Osaka, Japan) were applied, and the samples were incubated overnight at 4 °C. Cells were incubated with the corresponding secondary antibodies conjugated to Alexa 488 (Molecular Probe, Eugene, OR, 1:500) for 1 h at room temperature and mounted using fluorescence mounting medium containing 4′,6-diamidino-2-phenylindole (Dako, Glostrup, Denmark). Images were captured under an Olympus DP72 microscope equipped with a digital camera using Olympus DP2-Twain software. The antibodies used were as follows: anti-STAT1 (#14994, CST, 1:100), -STAT2 (#72604, CST, 1:100), -IRF9 (#76684, CST, 1:100), and -8-OHdG (#MOG-20P; JaICA, Shizuoka, Japan).

### Subcellular fractionation

The cytosolic and nucleic protein fractions were extracted stepwise using a ProteoExtract Subcellular Proteome Extraction Kit (Merck, Darmstadt, Germany) according to the manufacturer’s recommendations.

### Phos-tag gel analyses

Phos-tag, which involves determination of the mobility shift between phosphorylated and unphosphorylated proteins by SDS-PAGE,^11^ was performed to distinguish phosphorylated and unphosphorylated STAT1 and STAT2. A Mn^2+^-Phos-tag SDS-PAGE gel (Wako) was used in the assays according to the manufacturer’s instructions.

### Lentiviral transduction

JAK1-shRNA (#sc-35719-V), STAT1-shRNA (#sc-44123-V), and STAT2-shRNA (sc-29492-V) lentiviral particles were purchased from Santa Cruz Biotechnology (Santa Cruz, CA, USA). Cells were transduced with lentiviral particles and selected using puromycin.

### Plasmids, transfection, and dual luciferase assay

To determine ISRE activities, the ISRE-driven firefly luciferase-expressing reporter plasmid pISRE-luc (Clontech, Mountain View, CA), together with a pGL4-TK plasmid-expressing seapansy luciferase (Promega, Fitchburg, WI) as an internal control,^23^ were transiently transfected into the cells using FuGENE6 (Promega) according to the manufacturer’s protocol.^24^ After 48 h, dual luciferase assays were performed using a Dual Luciferase Reporter Assay System (Promega) as described previously.^25^

### Immunohistochemistry

Immunohistochemistry was performed as described previously.^26^ Endogenous peroxidase activity was blocked by incubation in 3% H_2_O_2_ for 30 min. Antigen retrieval was performed by incubating the slides at 89 °C in 10 mM sodium citrate buffer (pH 6.0) for 30 min. To minimize nonspecific background staining, slides were blocked in 5% normal goat serum (Dako) for 10 min at room temperature. Tissues were incubated overnight at 4 °C with the primary antibodies, followed by incubation with the corresponding HRP-conjugated secondary antibodies (Nichirei Bioscience, Tokyo, Japan) for 30 min. Probes were visualized with 3,3′-diaminobenzidine in buffered substrate (Nichirei Bioscience). Anti-STAT1 (#14994, CST, 1:500) and -STAT2 (#HPA018888; Sigma, St. Louis, MO; 1:500) antibodies were used. Tissue arrays containing human liver tissues (BC03116a) were purchased from US Biomax (Rockville, MD, USA).

### Immunoprecipitation

Immunoprecipitation was performed as described previously.^26^ In brief, cell lysates were mixed with anti-IRF9 antibodies and protein A magnetic FG beads (Tamagawa Seiki, Nagano, Japan) prewashed with immunoprecipitation buffer (50 mM Tris-HCl, pH 7.5, 150 mM NaCl, 0.1% NP-40, 1 mM ethylenediaminetetraacetic acid (EDTA), pH 8.0, 0.25% gelatin, and 0.02% sodium azide), and incubated with rotation overnight at 4 °C. The beads were then separated with a magnetic separator and mixed with 2× SDS sample buffer (0.25 M Tris-HCl, pH 6.8, 8% SDS, 40% glycerol, and 0.02% Bromophenol Blue) and boiled using a heat block at 95 °C for 3 min to elute the proteins. For western blotting, Trueblot anti-rabbit IgG HRP (1:1000; Rockland, Limerick, PA, USA) was used as the secondary antibody to avoid interfering with the immunoprecipitated immunoglobulin heavy and light chains.

### ChIP assay

To determine the binding of IRF9-related protein complexes to the promoter regions of ISGs, a ChIP assay was performed using a CST kit according to the manufacturer’s instructions. Briefly, 1× 10^7^ cells were processed according to the manufacturer’s protocol, and protein-DNA complexes were immunoprecipitated with 1 μg of anti-IRF9 antibodies. As a positive control, 10 μL of rabbit polyclonal anti-Histone H3 was used; as a negative control, 1 μL of normal rabbit IgG was used (both antibodies were included with the CST kit). PCR analysis of the immunoprecipitates was performed by a standard protocol using LA-Taq polymerase (Takara, Shiga, Japan), 3 μL of the appropriate DNA sample, and the following primers: *IFI27* (forward: 5′-CTTCTGGACTGCGCATGAGG-3′, reverse: 5′-CCACCCCGACTGAAGCACTG-3′) and *Mx1* (forward: 5′-GGGACAGGCA&CAACAAAGCC-3′, reverse: 5′-GCCCTCTCTTCTTCCAGGCAAC-3′).

### Statistical analysis

The significance of differences between groups was determined by Student’s *t* test when the variances were equal. When the variances were unequal, Welch’s *t* test was used. Values of *p* less than 0.05 were considered to indicate statistical significance.

## Electronic supplementary material

Supplementary Information

## Data Availability

The microarray data were deposited in the GEO database (accession number GSE107483). The data that support the findings of this study are available from the corresponding author upon reasonable request.

## Electronic supplementary material

**Supplementary information** accompanies the paper on the *npj Aging and Mechanisms of Disease* website (10.1038/s41514-018-0030-6).
