# Supplementary material for: ISGF3 with reduced phosphorylation is associated with constitutive expression of interferon-induced genes in aging cells
Source: NPJ Aging Mech Dis. 2018 Nov 15;4:11. doi: 10.1038/s41514-018-0030-6 (PMC6237867; doi:10.1038/s41514-018-0030-6)
Supplement: Supplementary file 1 — Supplementary Information [file 41514_2018_30_MOESM1_ESM.pdf]

Supplementary Information

ISGF3 with reduced phosphorylation is associated with constitutive expression of interferon-induced genes in aging cells

Mari Yamagami, Motoyuki Otsuka, Takahiro Kishikawa, Kazuma Sekiba, Takahiro Seimiya, Eri Tanaka, Tatsunori Suzuki, Rei Ishibashi, Motoko Ohno, and Kazuhiko Koike

Supplementary Information Inventory

Supplementary Figure Legends

Supplementary Figures S1 – S7

Supplementary Table

Supplementary Figure Legends

Supplementary Figure 1. The 8-OHdG-positive cells were increased in senescent NHDFs. 8-OHdG in NHDFs at early passage (p3) and senescent cells at passage 21 (p21) were stained by immunocytochemistry. Representative results from two independent experiments are shown. Bar, 10 μm. Positive nuclei were determined by counting 20 cells in every fifth field of view from two experiments in each group. Data are expressed as means ± s.e. $^{*}p < 0.05$.

Supplementary Figure 2. IFNα and IFNβ levels were increased in Huh7 cells stimulated with pIpC.

Huh7 cells were treated with 10 μg/mL pIpC for 24 h. IFNα and IFNβ levels were determined by qRT-PCR. Data are expressed as means ± s.e. of triplicate results from two independent experiments. *, $p < 0.05$.

Supplementary Figure 3. Unphosphorylated STAT levels were increased in ISGF3 complexes in senescent NHDFs.

IRF9 protein-related complexes in NHDFs at passage 8 (p8) and passage 23 (p23) were immunoprecipitated using anti-IRF9 antibodies, and the indicated proteins were immunoblotted. Normal rabbit IgG was used as a negative control (nc) for immunoprecipitation. IFNα-stimulated cells were used as a control for STAT protein phosphorylation. In all, 5% cell lysates were used as "input." Representative results from three independent experiments are shown.

Supplementary Figure 4. Increased ISGF3 complex binding to ISREs on target gene promoters.

After cross-linking with 1% formaldehyde and sonication, antibodies against IRF9 [for cells at early passage (p8) and senescent cells at passage 23 (p23)] or comparable amounts of antibodies against H3 and normal rabbit IgG (both for cells at p23) were used for a ChIP assay. Real-time PCR was performed to amplify the precipitated DNAs with primer pairs spanning ISREs in the promoters (*IFI27* and *Mx1*). The amounts of amplified DNA (% input) are the percentages of DNA in immunoprecipitated samples compared to 2% input DNA. All data are expressed as means ± s.e. of triplicate results from two independent experiments. $^{*}p < 0.05$, compared to the IgG control.

Supplementary Figure 5. ISRE promoter activities in Huh7 cells were increased by IFN treatment.

ISRE-driven luciferase expression constructs were transiently transfected into Huh7 cells, and a dual luciferase assay was performed after treatment with 100 U/mL IFNα for 24 h. nc, negative control. Data are expressed as means ± s.e. from two independent experiments performed in triplicate. *, $p < 0.05$.

Supplementary Figure 6. Uncropped images for the main figures.

Uncropped, full images of Western blotting for Figure 1c (a), Figure 3a (b), Figure 3b (c), Figure 3c (d), and Figure 3d (e).

Supplementary Figure 7. Uncropped images for the main figures.

Uncropped, full images of Western blotting for Figure 4b (a), Figure 4d (b), Figure 5c (c), Figure 5d (d), and Figure 5e (e).

Supplementary Figure 1

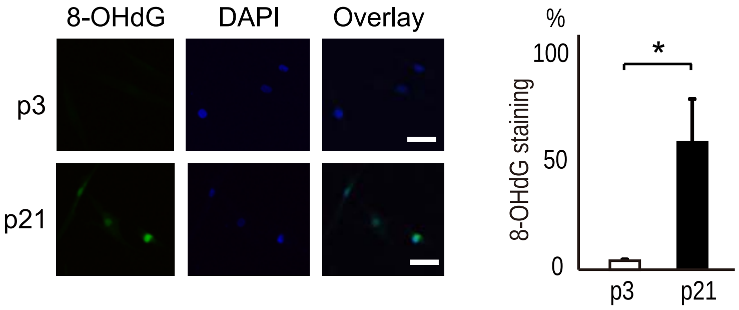

Supplementary Figure 2

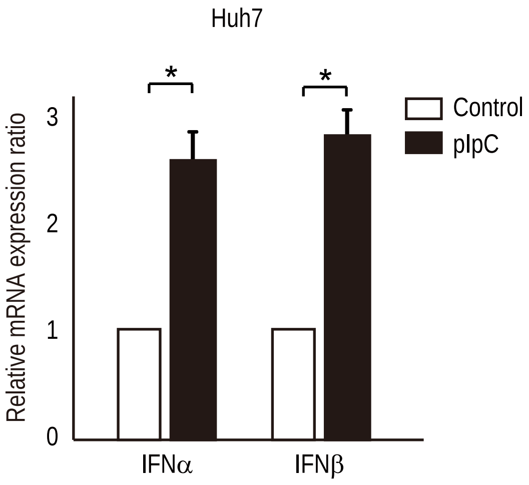

Supplementary Figure 3

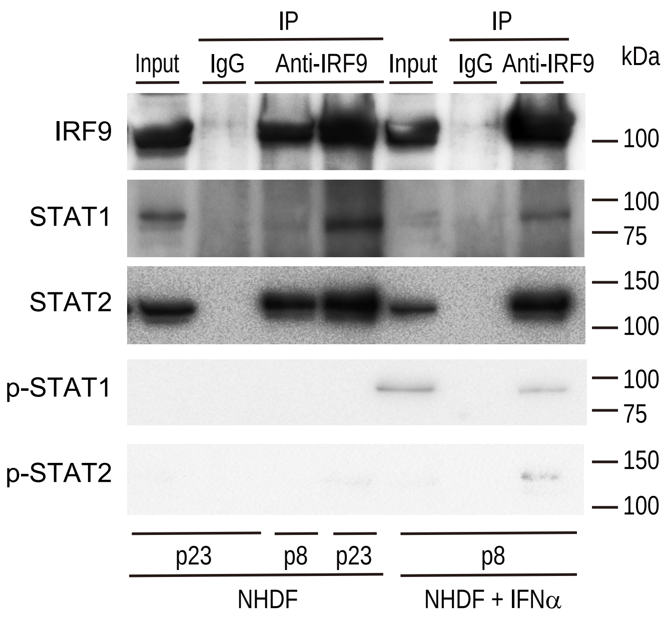

Supplementary Figure 4

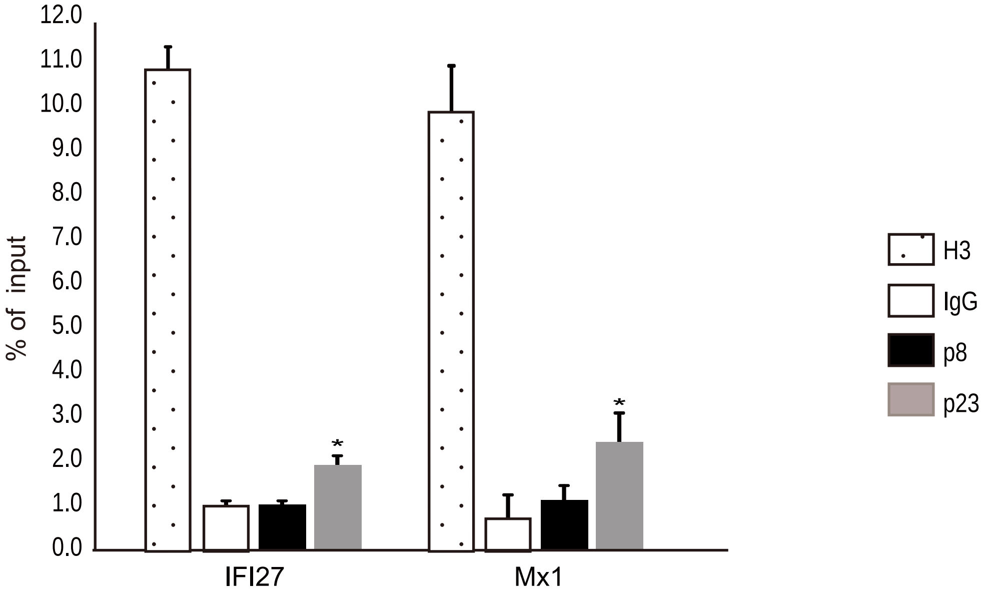

Supplementary Figure 5

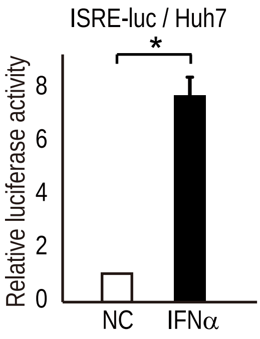

Supplementary Figure 6

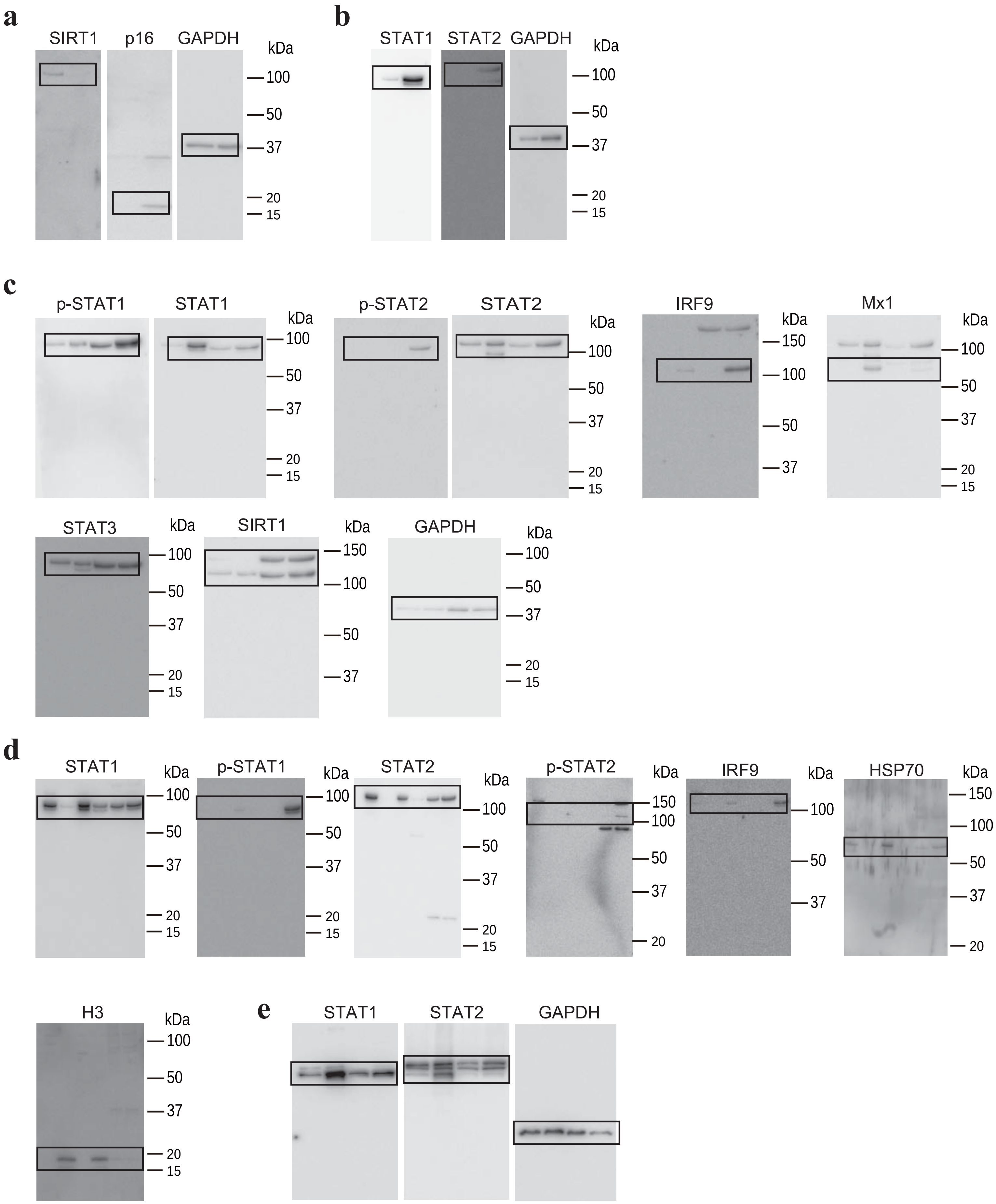

Supplementary Figure 7

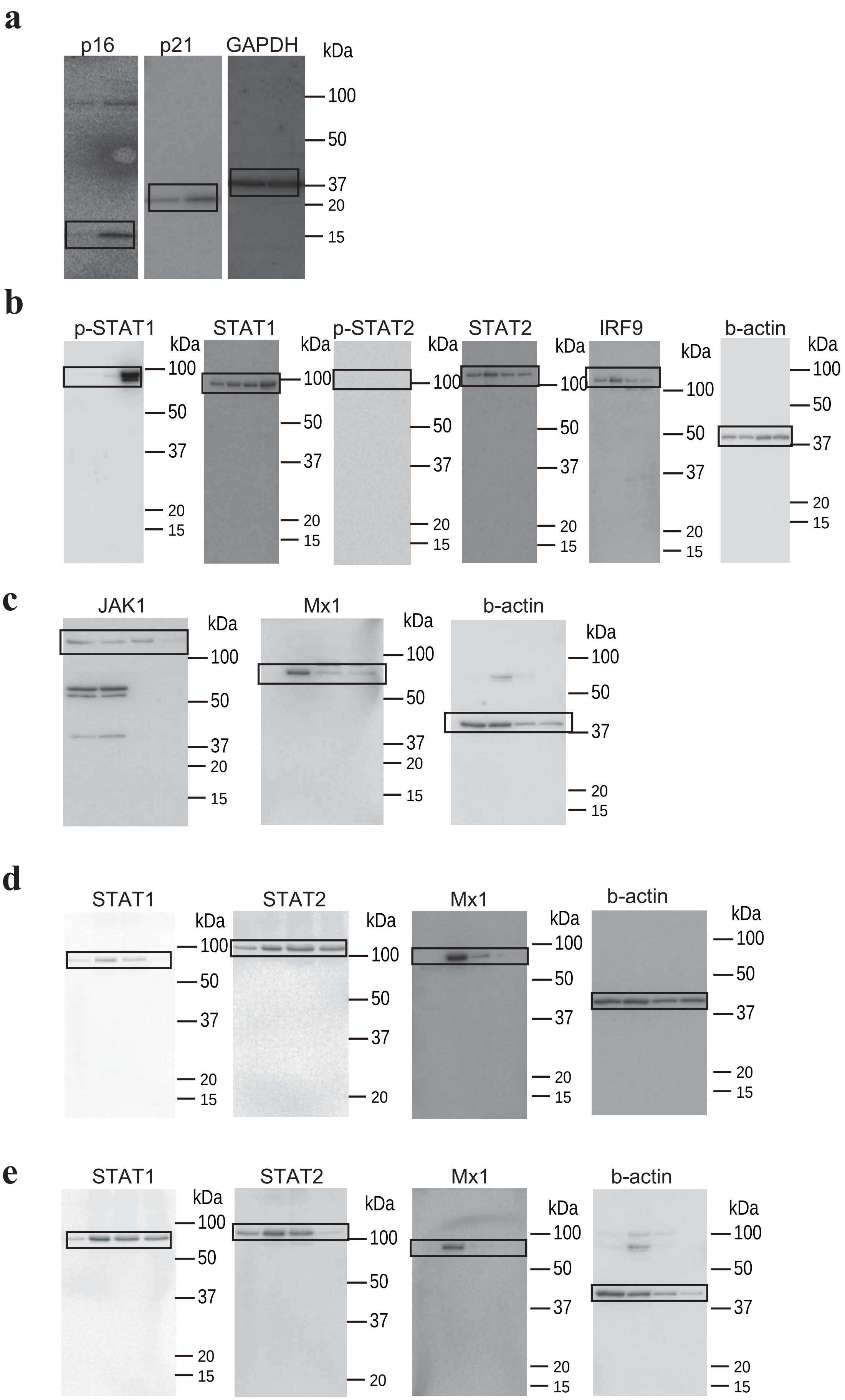

Gene	Description	Ratio
IL6	Interleukin 6	75.85
IFI44L	Interferon induced protein 44 like	48.63
ITM1	Interferon induced transmembrane protein 1	41.54
OASL	2'-5'-oligoadenylate synthetase-like	29.62
MX2	MX dynamin-like GTPase 2	22.86
OAS1	2'-5'-oligoadenylate synthetase 1	22.44
ITIH5	Inter-alpha-trypsin inhibitor heavy chain family member 5	21.19
MX1	MX dynamin like GTPase 1	19.30
ISG15	ISG15 ubiquitin-like modifier	17.88
IFI6	Interferon, alpha-inducible protein 6	16.11
IFIT3	Interferon induced protein with tetratricopeptide repeats 3	15.57
ITIH5	Inter-alpha-trypsin inhibitor heavy chain family member 5	14.26
IFIT1	Interferon induced protein with tetratricopeptide repeats 1	14.05
OAS3	2'-5'-oligoadenylate synthetase 3	14.00
IFI27	Interferon, alpha-inducible protein 27	11.94
STAT1	Signal transducer and activator of transcription 1	5.83
STAT2	Signal transducer and activator of transcription 2	2.90
STAT3	Signal transducer and activator of transcription 3	1.13
IFNA1	Interferon, alpha 1	1.12
IFNB1	Interferon, beta 1, fibroblast	1.22
IFNG	Interferon, gamma	0.78

Supplementary Table. Results of the representative IFN-induced genes from cDNA analyses.

Ratio indicates the fold changes of the gene expression levels in the older NHDFs compared with the younger cells. IL6, in the top column, is a representative SASP. Second column categorizes IFN-stimulated genes. Third column categorizes STAT genes. The fourth column categorizes IFN family genes.
